# Medical care epidemiology and unwarranted variation: the Israeli case

**DOI:** 10.1186/s13584-017-0135-6

**Published:** 2017-02-20

**Authors:** David C. Goodman, Andrew A. Goodman

**Affiliations:** 1 The Geisel School of Medicine at Dartmouth, The Williamson Translational Research Building, The Dartmouth Institute for Health Policy and Clinical Improvement, Lebanon, 03756 NH USA; 2 Goodman Health Services, 65 Whitney Farms Rd, North Yarmouth, 04097 ME USA

## Abstract

In an article in this Journal, Mendlovic and colleagues report on regional variation in medical care across Israeli regions. This study joins a growing literature demonstrating generally high variation in the provision of health care services within developed countries. This commentary summarizes the status of medical care epidemiology and its studies of unwarranted variation in health care, and provides a conceptual framework to guide future studies. Recommendations are offered for advancing studies in Israel that could guide policy development and clinical improvement.

## Background

If we want to improve health care, systematic population-based measurement of health care delivery is essential [1]. While the importance of ascertaining population health status is well recognized, medical care as a focus of measurement is less developed. Barriers include poor data availability and the reluctance to publicly report findings, resulting in a failure to identify many opportunities for policy development and clinical improvement. While there are a few countries in which medical care epidemiology is robust, the implementation of this essential partner to disease epidemiology remains uneven [2]. Researchers outside the U.S., U.K., and Canada have been relatively slow in initiating analysis of medical care and its variation until a decade ago, when academic and governmental interest began to grow. One example of greater national interest is Israel’s participation in the medical practice variation project of the Organisation for Economic Co-operation and Development (OECD) [3] which led to Mendlovic and colleagues’ recent paper in IJHPR [4]. This article reports an important study for the Israeli health care system and makes a notable contribution to the international effort to better understand health care and target improvement.

The landscape of Israeli hospital utilization shows striking similarities, and some differences, to the other 12 countries participating in the OECD project [3]. With two exceptions, regional variation of medical hospitalizations and procedures was markedly high, a phenomenon observed for most types of medical and surgical care wherever measured [5]. Low variation in utilization was found for surgery after hip fracture in Israel as has also been observed in other developed countries. This can be attribiuted to professional certainty in diagnosis and the need for treatment. Almost all hip fractures are diagnosed and have surgery. Therefore, hip fracture with surgery rates correlate closely with disease incidence. In general, differences in populations, including health status, are less important contributors to variation than is professional uncertainty and physician practice styles. More unusual was Israel’s low variation in hysterectomies, but Israel also has the lowest rate of hysterectomies of the 13 reporting OECD countries. To place Israel’s rate of 122 hysterectomies per 100,000 in perspective, Spain had the next highest rate at 172, and Canada’s rate was 394, the highest of the 13 participating countries.

## Development of medical care epidemiology

Description is the start of any epidemiologic analysis, and quickly leads to questions of causation. What factors lead to high medical practice variation? Can we say that some types of medical practice variation are “good” and other types “bad?” If we determine the causes, are there remedies that might improve health care and health? At this early stage, there are no specific answers to these questions for Israel. Inferential analysis of the causes and health consequences of variation proceeds much more slowly than description. But there is a sizable body of inferential research from other countries that may have relevance to Israel [5].

The primary motivation to study patterns of health care utilization is to gain insight into the performance of health care providers and systems. Suboptimal health care performance has consequences for population health, but variation in health care is only partially explained by area population differences. The technical term for the variation that is not explained by patient needs or preferences is “unwarranted variation” and reflects differences in health care quality and efficiency. Originally, analyses examining unwarranted variation were termed “small area analysis,” [6] in reference to analysis across empirically defined health care service *areas* (i.e. geographic health care markets). With improvements in data quality, recent efforts have been directed toward the measurement across *providers*, such as hospitals. Regardless of the units that define the population or patient denominators, the study of health care variation faces similar challenges in methods and interpretation.

While the first study of medical practice variation was published in England in 1938, [7] John Wennberg’s 1973 paper in *Science* reporting differences in health care resources and utilization across the relatively homogenous State of Vermont marked the beginning of rapid growth in small area analysis studies in the U.S. [8]. Given today’s recognition of the uneven shortcomings in health care, it may be difficult to appreciate that in the 1970s these findings were initially ignored, later attacked, [9] before being replicated [10–12] and then widely embraced by clinicians, health system administrators and policy makers in the U.S. Canada, and the U.K. A notable testament to the seminal character of Wennberg’s paper is its citation by over 1200 other academic papers [13]. Subsequent studies by Dr. Wennberg and his Dartmouth colleagues further advanced the methods to interpret regional patterns of care from the beginning to the end of life [14, 15]. Cohort studies were developed to address inherent weaknesses in cross-sectional designs, and ecologic analyses were supplanted by multi-level models with the patient as the unit of analysis [16–18]. With funding from the Robert Wood Johnson Foundation in 1992, Wennberg and colleagues introduced the *Dartmouth Atlas of Health Care* series (see www.dartmouthatlas.org) as a dissemination tool directed towards non-academic audiences, such as health care administrators, health policy makers, and congressional staffers. At the same time, research into health care variation grew in the U.K. and Canada, and more recently, with strong support from national and provincial health ministries [5]. In the past 40 years, these and other studies of unwarranted variation have influenced the practice of medicine, the organization of delivery systems, the financing of medical care, and national health care policy.

## Types of variation

The most widely used framework for interpreting medical practice variation parses unwarranted variation into three categories—effective, preference sensitive, and supply sensitive care.

### Variation in effective care

Variation in effective care reflects differences in technical quality, where medical care interventions have high benefit and low risk. Usually, the “right” rate is close to 100% for the target population. Obvious examples are childhood immunizations, the use of beta blockers and aspirin in myocardial infarctions, and monitoring HgA1C in diabetics. Low utilization rates indicate unwarranted variation in the provision of effective medical practice. Reducing variation in effective care is the most common focus of clinical quality improvement efforts in Israel [19] and internationally. Technical assistance in improving effective care is available from the Institute for Health Care Improvement or the International Society for Quality in Healthcare [20, 21].

### Variation in preference-based care

Preference-based care refers to utilization variation for decisions where there are more than one diagnostic and therapeutic option, each with its own profile of benefits and harm. The original analyses that led to this concept were studies of the treatment of benign prostatic hypertrophy in adult men [22]. In the early 1980s, the high regional variation in the use of transurethral prostatectomies in the U.S. was driven by local differences in theories of benefit and harm held by urologists. Patients usually followed their physician recommendations, which differed across urologists. The risk/benefit profile of TURP compared to watchful waiting was based on a poor evidence base, but in the late 1980s effectiveness studies showed that TURPs offered the benefit of easier and less frequent urination but with the tradeoff of much higher rates of incontinence and sexual dysfunction than previously appreciated by the medical community. Most importantly, average patience preferences favored less TURPs than were observed in utilization rates. These average preferences hid the diversity of values (i.e. utilities) assigned by patients and physicians to the known outcomes. While the “best” choice was the decision that would maximize the utility for each patient, most decisions rested on the average utility assigned by the urologist. Examples of preference sensitive care include treatment options associated with early stage prostate and breast cancer, lower back pain, and arthroplasty. Variation in treatment utilization rates for these conditions reflects the local practice style of physicians, which originates from training and then is molded by clinical experience.

Unfortunately, physicians are poor at diagnosing patient preferences [23]. Improving decision quality through shared decision-making requires new methods of conveying information to patients, such as decision aids, [24] accompanied by encouraging patient identification of preferences and participation in the decision process. Often, but not always, the introduction of decision aids reduces utilization rates of procedures or aggressive care. Unlike effective sensitive care, there is no single “right rate.” The right rate for a population reflects the average decisions of informed and engaged patients and families. It is expected that the care choices may differ across families, and in turn, across regions.

### Variation in supply sensitive care

Supply sensitive care refers to medical services for which utilization rates are responsive to the local availability of health care capacity, such as beds and physicians [25]. These services have also been termed discretionary. While in some instances a low supply of resources may constrain effective care, this category is concerned with the range of capacity that is viewed as adequate.

The backdrop to supply sensitive care is that the regional variation in capacity in many countries does not reflect differences in population need. Despite some efforts to direct physicians, hospital beds, and catheterization labs to places where health status is lower, historical patterns of hospital building and physician location patterns tend to favor more affluent and attractive communities [26, 27].

In the U.S., medical admissions for chronic illness are considered supply sensitive care [28, 29]. Medical discharge rates in the elderly vary 200–300% across hospital services areas and are strongly influenced by area bed supply [26]. How can this occur? Congestive heart failure (CHF) hospitalizations are a useful example. A clinician caring for a patient in the office or ER with signs of worsening CHF has the option of initiating more aggressive treatment with outpatient monitoringor hospitalization, and will decide after considering physiologic parameters, past medical history, and available family and patient preferences. Clinical opinions regarding the “right choice” for a specific patient can differ frequently, even for physicians familiar with clinical guidelines. Invisible to the clinician is the local supply of beds and its subtle influence on the threshold of admission. As bed supply varies, so does discharge rates for CHF along with most other medical causes of admission. There is no evidence that U.S. patients are harmed in regions with lower bed supply and medical hospitalization rates, suggesting that there are unrealized efficiency gains in reducing unnecessary acute care [16, 17].

Further examples of supply sensitive care include the number of hospital or ICU days for medical care, emergency room visits, rates of consultations, physician revisit rates, and imaging procedures. The decisions related to these medical care events elude the concepts of efficacy and effectiveness. Unlike effective care, population rate differences are very weakly associated with outcomes [16, 17].

## Israeli medical practice variation in context

Caution is needed in applying these ideas to Israel’s patterns of care. The old adage comes to mind: if you’ve seen one health care system, you’ve seen one health care system. At the same time, the experiences from the U.S. and from other OECD countries may identify future directions for Israeli health policy research. Several recommendations follow:

Measuring health across regions and providers requires consistent efforts at improving measurement, and encouraging inquiry by researchers and policy analysts. Just as disease epidemiology continually monitors and investigates health and disease, medical care epidemiology requires a national commitment to using the findings of population-based evidence to improve care. Israeli patients would benefit from an expansion of health care measures that are tabulated annually. Also, allowing researchers access to the data would bring new insights into the delivery of medical care.

Studies should incorporate more recent methods for controlling for population differences to better reveal the proportion of variation that is unwarranted [5]. The best method is highly dependent on the specific research question, the population of interest, and the quality of available data. Applying improved methods of population adjustment would increase the inferential value of the results.

Given the heterogeneity of both population and health care delivery in Israel, further insight may be gained by studies that use smaller areas or hospitals as the units of analysis. Ideally, the scale of measurement is dictated by the extent of regionalization of care, ranging from primary care [30] to tertiary care [31]. Hospital specific cohorts for conditions such as acute myocardial infarctions or end of life cancer care can provide high specificity and insight into the responsible health care systems [32, 33].

Finally, without identifying the causes of unwarranted variation, improvement efforts operate in the dark. As descriptions of the Israeli health care utilization develop, a national investment in research examining the causes and consequences of variation is likely to have high returns for patients.

## Conclusion

The strains in health care are increasingly evident. The public expects continual gains in medical science, in the development of prevention measures and illness treatment, and in their equitable dissemination through an ever more comprehensive health system. Care known to have high benefits to patients is often incompletely implemented. Low value care disguised as innovative treatments frequently takes root, often at great expense and potential harm. In some instances, patients remain in the dark about available treatment options, as clinicians make well-meaning recommendations that align poorly with patient preferences. Worldwide, rising costs challenge the ability of the health care system to meet these expectations as health care takes an increasing proportion of national wealth, competing against needs in education, housing, and other strategic investments. Israel has successfully constrained spending as a proportion of GDP, but the growth of private spending is high [34]. As public expectations continue to rise in an era of limits in public and private spending, measuring health system performance and its variation across providers can guide the search for better value in health care.
